# Country-specific estimates of misclassification rates of computer-coded verbal autopsy algorithms

**DOI:** 10.1136/bmjgh-2025-021747

**Published:** 2026-03-09

**Authors:** Sandipan Pramanik, Emily Wilson, Henry D Kalter, Victor Akelo, Agbessi Amouzou, Robert Black, Dianna Blau, Ivalda Macicame, Jonathan A Muir, Kyu Han Lee, Li Liu, Cynthia G Whitney, Scott Zeger, Abhirup Datta

**Affiliations:** 1 Department of Biostatistics, Johns Hopkins University, Baltimore, Maryland, USA; 2 Department of International Health, Johns Hopkins University, Baltimore, Maryland, USA; 3 Centers for Disease Control and Prevention Global Health Kenya, Kisumu, Kenya; 4 Global Health Center, Centers for Disease Control and Prevention, Atlanta, Georgia, USA; 5 Instituto Nacional de Saúde, Maputo, Mozambique; 6 Emory Global Health Institute, Emory University, Atlanta, Georgia, USA; 7 Department of Population, Family and Reproductive Health, Johns Hopkins Bloomberg School of Public Health, Baltimore, Maryland, USA

**Keywords:** Global Health, Child health, Mathematical modelling

## Abstract

**Introduction:**

Computer-coded verbal autopsy (CCVA) algorithms are routinely used to determine individual cause of death (COD) and derive population-level estimates of cause-specific mortality fractions (CSMFs). But frequent COD misclassification leads to biased CSMF estimates. The VA-calibration framework reduces the bias by estimating misclassification rates; but it overlooks systematic patterns and cross-country variation, reducing the accuracy of CSMF estimates.

**Methods:**

Using CHAMPS (Child Health and Mortality Prevention Surveillance) data and the framework in Pramanik *et al (2025)*, we estimate misclassification rates of three widely used CCVA algorithms (Expert Algorithm VA, InSilicoVA and InterVA), two age groups (neonates aged 0–27 days and children aged 1–59 months), and eight countries (Bangladesh, Ethiopia, Kenya, Mali, Mozambique, Sierra Leone, South Africa and ‘other’). We then demonstrate their utility and use the Mozambique-specific rates to calibrate VA-only data from the Countrywide Mortality Surveillance for Action (COMSA) project in Mozambique.

**Results:**

We report three key findings. First, the country-specific model better fits CHAMPS misclassification rates than the homogeneous model, reducing average absolute loss by 34%–38% for neonates and 13%–24% for children. Second, CCVA algorithms show consistent misclassification patterns, systematically overestimating or underestimating certain causes. Third, calibrating COMSA data increases neonatal CSMF for sepsis/meningitis/infection and decreases it for intrapartum-related events and prematurity; among children, CSMF increases for malaria and decreases for pneumonia.

**Conclusions:**

We present an inventory of VA misclassification rate estimates across two age groups, three CCVA algorithms and eight countries. These publicly available estimates enable the calibration of VA-only data from any country without needing access to CHAMPS data. More generally, these analyses reveal systematic algorithmic biases and highlight opportunities to refine future CCVA algorithms. As reliance on computer-coded and AI-driven approaches to COD determination grows, our integrated VA-calibration workflow, grounded in robust statistical frameworks and open-source software (misclassification matrix modeling, VA-calibration R package on GitHub and CRAN), offers a critical step towards improving the accuracy of mortality surveillance.

**WHAT IS ALREADY KNOWN ON THIS TOPIC**

- CCVA algorithms are widely used to estimate individual-level COD, which are frequently misclassified.
- The misclassification leads to biased CSMF estimates.
- Existing VA-calibration methods^26 27^ account for misclassification using limited labelled COD data, such as from the CHAMPS Network, but they do not address the underlying systematic patterns and significant cross-country variation.

**WHAT THIS STUDY ADDS**

- We use the misclassification matrix modelling framework in Pramanik *et al*^30^ and produce an inventory of uncertainty-quantified, country-specific misclassification estimates for three widely used CCVA algorithms (Expert Algorithm VA, InSilicoVA, and InterVA), two age groups (neonates aged 0–27 days and children aged 1–59 months), and eight countries (Bangladesh, Ethiopia, Kenya, Mali, Mozambique, Sierra Leone, South Africa, and ‘other’).
- We demonstrate their utility by applying Mozambique-specific estimates to VA-only data from the COMSA project in Mozambique, improving national-level CSMF estimates for neonates and children.

**HOW THIS STUDY MIGHT AFFECT RESEARCH, PRACTICE OR POLICY**

- We publicly share the inventory of misclassification estimates obtained here to support calibration of VA-only data worldwide.
- The analysis uncovers systematic algorithmic biases, indicating opportunities where future algorithm performance can be improved.
- Amid ongoing advances in artificial intelligence (AI) for COD determination, the increasing reliance on computer-coded algorithms and their inherent risk of misclassification, the integrated VA-calibration workflow, supported by statistical frameworks and software (misclassification matrix modeling, VA-calibration R package on GitHub and CRAN), represents a crucial step towards enhancing the accuracy of algorithm-based and AI-driven mortality surveillance.

## Introduction

Conventional cause-of-death (COD) diagnostic procedures, like full or limited autopsies, are often challenging to implement in low- and middle-income countries (LMICs) due to their resource-intensive nature.^1 2^ Verbal autopsy (VA) is a less invasive alternative for estimating COD in community settings.^3^ VA systematically interviews caregivers using a WHO-standardised questionnaire and gathers reports of the decedents’ illness signs, symptoms and available health records. The survey responses are either reviewed by physicians or processed using computer-coded VA (CCVA) algorithms to estimate a COD (VA-COD).

Many specialised CCVA algorithms have been developed to predict COD from VA records; for example, Expert Algorithm VA (EAVA),^4^ InSilicoVA,^5^ InterVA,^6^ Tariff,^7^ the King and Lu method,^8^ and domain adaptation-based methods.^9 10^ The openVA R package integrates many of these tools into one software.^11 12^ Generic classifiers such as random forests,^13^ naive Bayes classifiers^14^ and support vector machines^15^ have also been employed.^7 16 17^ Due to its minimally invasive nature and scalability enabled by CCVA algorithms, VA is being widely used to predict COD and build nationally representative VA-COD databases in many countries. Examples include the Countrywide Mortality Surveillance for Action (COMSA) programmes in Mozambique (COMSA-Mozambique) and Sierra Leone.^18^,^20^ Individual-level VA-COD data are often naively aggregated to estimate age-specific national and subnational cause-specific mortality fractions (CSMFs), the proportion of deaths in each age group due to specific causes.^21^,^24^ These efforts contribute to achieving health-related Sustainable Development Goals.^25^

Although CCVA algorithms are essential for large-scale mortality surveillance, they often misclassify COD compared with medical certification, full autopsy, or minimally invasive tissue sampling (MITS). This leads to biased CSMF estimates. VA-calibration addresses this by using paired VA and ‘gold standard’ (eg, MITS) data to estimate misclassification rates and improve CSMF estimation accuracy.^26 27^ This approach was recently applied in Mozambique, combining VA-only data from COMSA-Mozambique with limited MITS-VA data from the Child Health and Mortality Prevention Surveillance (CHAMPS) project.^19 28 29^

To counteract limited CHAMPS data, VA-calibration originally pooled data across countries, assuming uniform misclassification rates. While this improved precision, recent findings reveal significant cross-country variation, challenging that assumption.^30^ To address this, Pramanik *et al*^30^ proposed a country-specific misclassification matrix modelling framework that models global (shared) patterns underlying misclassification, mitigates limited samples and improves CSMF estimation accuracy.

We make two key contributions. First, we apply the framework to CHAMPS data and estimate uncertainty-quantified country-specific misclassification rates for three CCVA algorithms (EAVA (deterministic), InSilicoVA and InterVA (both Bayesian)), two age groups (neonates aged 0–27 days and children aged 1–59 months), and eight countries (Bangladesh, Ethiopia, Kenya, Mali, Mozambique, Sierra Leone, South Africa, and ‘other’). Second, it identifies systematic biases in each algorithm’s COD prediction, revealing previously unknown aspects of their functioning and informing future refinements. We demonstrate the value of country-specific misclassification estimates by calibrating VA-only COD data from COMSA-Mozambique and obtaining more accurate CSMF estimates.

## Data

### COMSA-Mozambique VA data

The COMSA-Mozambique was motivated by an interest in determining CODs across all age groups using representative samples.^19^ Provincially representative pregnancy and mortality data were collected through routine community surveillance in 700 clusters, each with around 300 households. Deaths were recorded at the community level, followed by interviews with caregivers using an integrated verbal and social autopsy questionnaire based on the 2016 WHO VA instrument. Currently, misclassification rates for VA can only be estimated for neonates and children aged under 5 years, due to the availability of CHAMPS data for these age groups. As such, our analysis of VA data from COMSA-Mozambique is limited to these populations, including 1192 neonatal (aged 0–27 days) and 2812 child (aged 1–59 months) records from January 2018 to December 2023.

Predicted CODs from EAVA, InSilicoVA, and InterVA were grouped into six broad categories for neonates and nine for children. Neonatal causes included congenital malformation, pneumonia, sepsis/meningitis/infections, intrapartum-related events (IPRE), prematurity and ‘other’. COD in children encompassed malaria, pneumonia, diarrhoea, severe malnutrition, HIV/AIDS, injury, neonatal causes (IPRE, congenital malformation and prematurity), other infections, and ‘other’.

### CHAMPS data

The CHAMPS Network collects premortem clinical and laboratory records, as well as postmortem VA and MITS from sites in Bangladesh, Ethiopia, Kenya, Mali, Mozambique, Sierra Leone and South Africa. At CHAMPS surveillance sites, a rapid mortality notification system ensures that all deaths among children aged under 5 years and stillbirths are reported to local teams within 24 hours. Informed consent for eligible cases is obtained from the parents or guardians. CODs are then investigated using MITS alongside a suite of laboratory analyses (typically taking 4 months), including microbiological and histopathological testing as well as diagnostic testing for HIV, tuberculosis, malaria and other infectious agents. Caregiver interviews conducted via VA are also requested to collect detailed symptom histories and contextual clinical information. A multidisciplinary Determination of COD (DeCoDe) panel then reviews all collected data and ascertains the COD, which is subsequently communicated to the family. Where multiple causes are present, the DeCoDe panel determines the causal chain, including the primary or underlying as well as the secondary or immediate and intermediate causes leading to death. Here, we include only the primary cause (*CHAMPS cause* from here on) for 1379 neonatal records and 1080 records for children (see online supplemental figures S1 and S2 for neonates and online supplemental figures S14 and S15 for children for a description). All deaths that occurred between December 2016 and June 2023 were grouped into the broad causes mentioned above. To analyse the accuracy of CCVA algorithms, we use the CHAMPS cause determined by the DeCoDe panel as the gold standard or reference COD.

## Methods

### VA-calibration and heterogeneous misclassification

For VA-only studies like COMSA-Mozambique, the *raw* or *uncalibrated* CSMF *q*_*j*_ for cause *j* is estimated as

.$${\hat{q}}_{j}=\frac{\text{Number of VA-predicted deaths from cause }j}{\text{Total number of VA records for that age group}}$$

Under misclassification, they differ from their true values according to the calibration equation $q_{j}=\sum_{i=1}^{C}\phi_{ij}p_{i}$.^26 27^ Here, *C* is the total number of causes, $\Phi =(\phi_{ij})$ is the *C*×*C* misclassification matrix, $\phi_{ij}$ is the rate at which the algorithm classifies CHAMPS cause *i* as cause *j* (the diagonals $\phi_{ii}$ are sensitivities, and the off-diagonals $\phi_{ij}$ are false negatives), and *p*_*i*_ is the true CSMF of cause *i* (with CHAMPS cause assumed as the truth). While uncalibrated estimates appear more precise, they ignore misclassification as evidenced in CHAMPS, risking biased and overconfident results. Leveraging limited paired CHAMPS-VA COD data, VA-calibration solves an inverse problem to correct for this bias. It produces a *calibrated CSMF estimate*, which increases uncertainty but improves out-of-sample predictive performance (see Datta *et al,*^26^ Fiksel *et al,*^27^ and Section 4 in Pramanik *et al*^30^).

**Figure 1:**
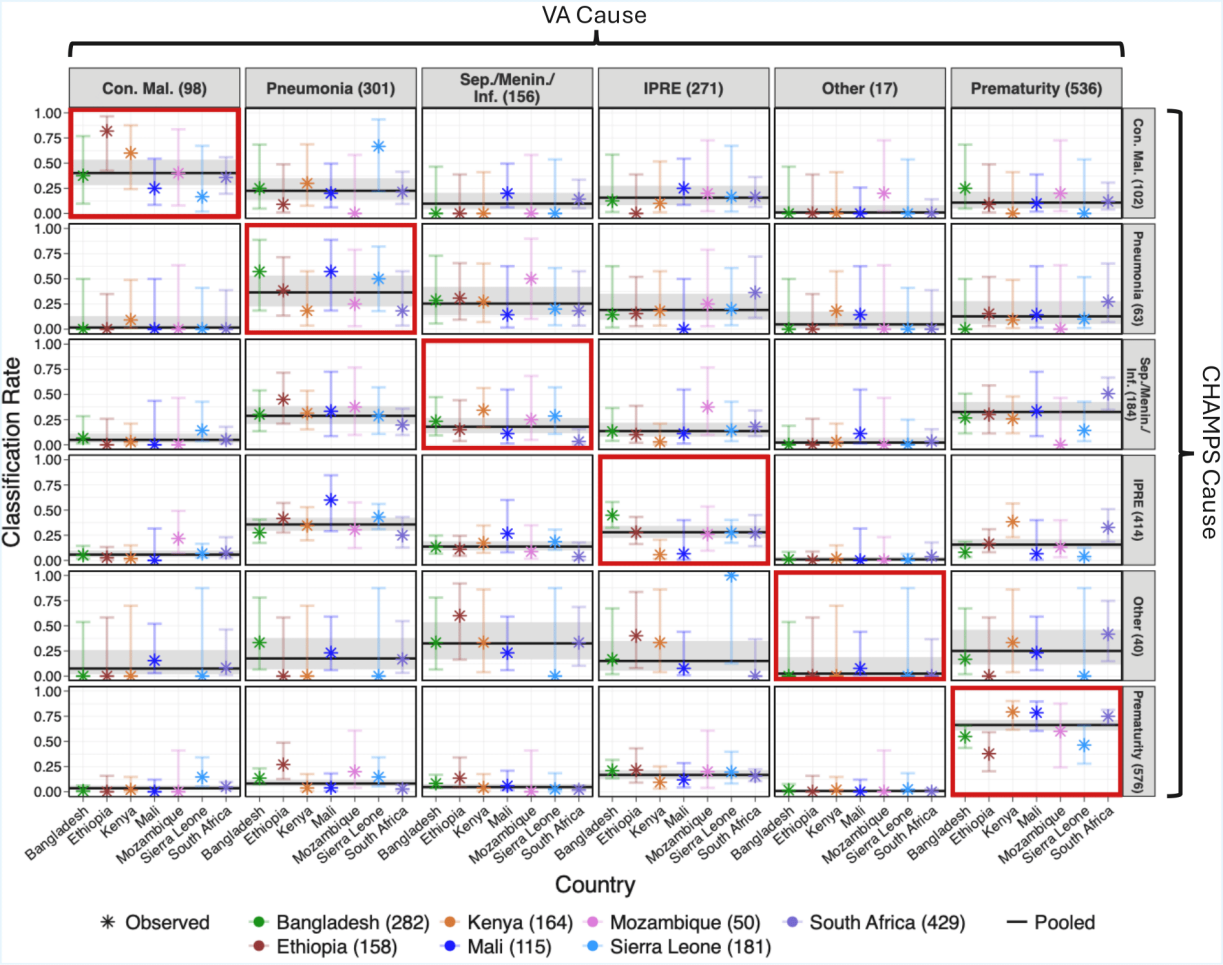
Observed misclassification rates of EAVA for neonates (aged 0–27 days) in CHAMPS. Rows and columns indicate CHAMPS and VA causes, with combined sample sizes across countries indicated in parentheses. Sensitivities and false negatives are shown along the diagonal (red) and off-diagonal panels, respectively. Misclassification rates are conditioned on CHAMPS cause (row), so values in each row sum to one for each country. CHAMPS, Child Health and Mortality Prevention Surveillance; Con mal, congenital malformation; EAVA, Expert Algorithm VA; IPRE, intrapartum-related events; Sep/Menin/Inf, sepsis/meningitis/infection; VA, verbal autopsy.

The current VA-calibration approach pools CHAMPS data across countries to improve sample size and precision in estimating misclassification rates. Pooling assumes the same VA misclassification across countries, but its effectiveness relies on how well they represent the study country.^27^ While this assumption cannot be tested in VA-only settings due to the unavailability of CHAMPS-COD, it rests on the idea of globally similar symptom-cause relationships. In contrast, CHAMPS data show significant cross-country variation in misclassification rates (eg, see figure 1 for EAVA and online supplemental figure S3), potentially biasing CSMF estimates.

### Modelling structure and heterogeneity in VA misclassification

Pramanik *et al*^30^ proposed an efficient Bayesian modelling framework that improves precision in country-specific VA misclassification estimation under limited samples (see online supplemental sections S1.1–S1.5). The framework incorporates the following key components:

1. They introduce a parsimonious base model based on two underlying latent mechanisms, *intrinsic accuracy* and *pull*, that characterise global misclassification patterns. Intrinsic accuracy reflects the algorithm’s ability, by design, to correctly identify a true cause. When it fails to correctly identify the true cause by design, Pull captures systematic bias, indicating an algorithm’s tendency to overpredict or underpredict certain causes regardless of the true cause (online supplemental figures S6 and S24). Together, they promote model parsimony, improving efficiency under limited samples. The framework builds on this and extends to country-specific modelling.
2. The framework adaptively chooses its complexity based on the data using a continuous shrinkage. This balances the bias-variance trade-off, favouring simpler models under limited samples or the absence of evidence.
3. The model produces uncertainty-quantified, country-specific misclassification estimates for seven CHAMPS countries, along with an estimate for countries outside CHAMPS. The estimate for other countries is centred on the pooled rate, with uncertainty reflecting the degree of homogeneity within CHAMPS. This enables VA-calibration in any country without requiring direct access to CHAMPS data. Additionally, estimates of intrinsic accuracy and pull reveal new insights into how CCVA algorithms function.

The framework improves VA misclassification estimates, leading to more accurate CSMF estimates when calibrating VA-only data like in COMSA-Mozambique.

### Modular VA-calibration using country-specific VA misclassification

#### Algorithm-specific calibration

**Figure 2:**
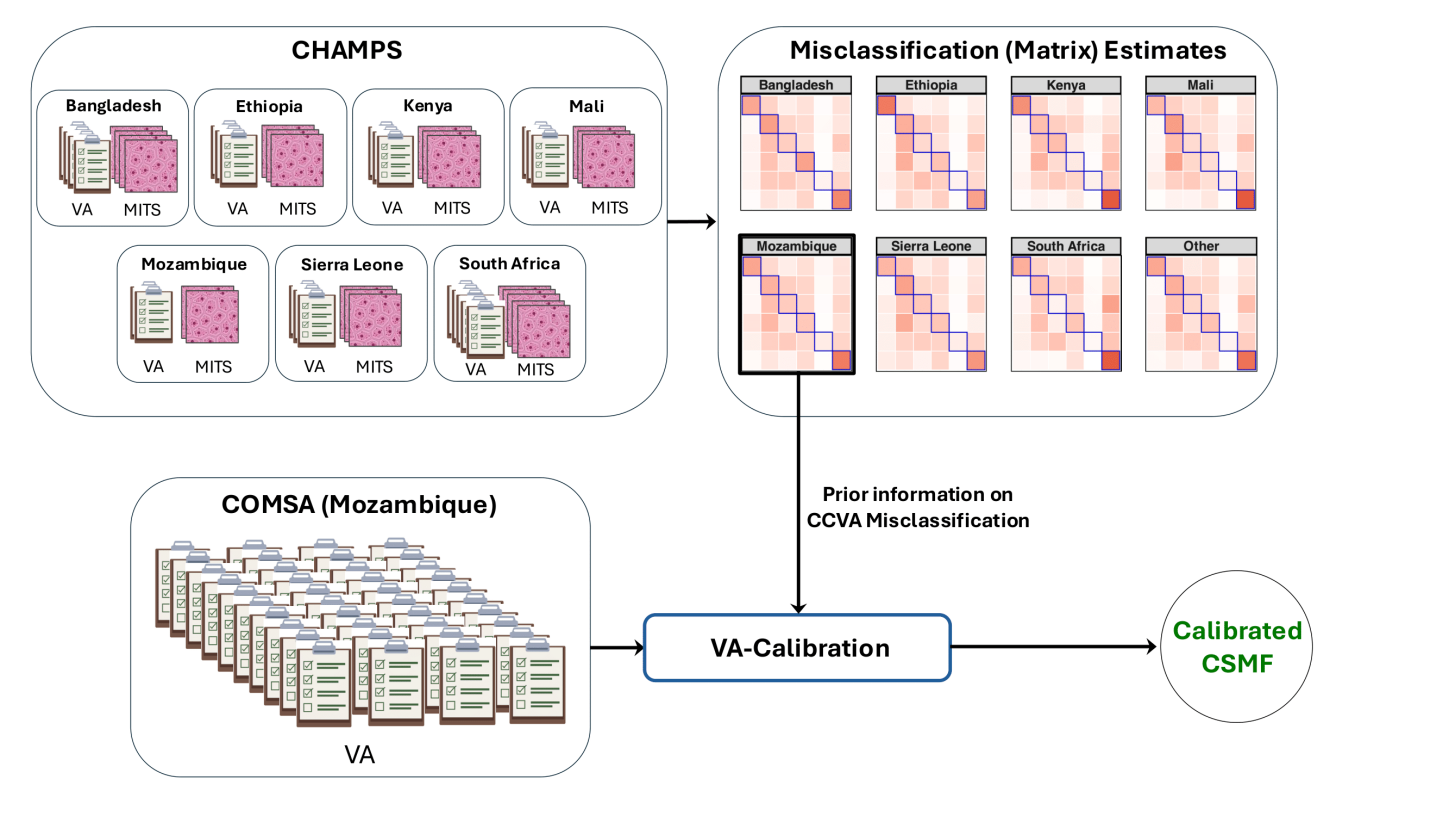
The modular VA-calibration pipeline for generating calibrated CSMF estimates from VA studies such as the COMSA in Mozambique. Country-specific misclassification estimates at the top are obtained using limited paired COD diagnoses from MITS and VA collected through the CHAMPS project. CCVA, computer-coded VA; CHAMPS, Child Health and Mortality Prevention Surveillance; COD, cause of death; COMSA, Countrywide Mortality Surveillance for Action; CSMF, cause-specific mortality fraction; MITS, minimally invasive tissue sampling; VA, verbal autopsy.

The current VA-calibration approach jointly models CHAMPS and VA-only data, requiring access to both.^28^ We apply the modular VA-calibration from Pramanik *et al*.^30^ This analyses the CHAMPS data once using the framework in Pramanik *et al.*^30^ and stores uncertainty-quantified estimates of misclassification matrices, resolved by country, age group and CCVA algorithm. To calibrate for a VA-only study like COMSA-Mozambique, country-specific, age-specific and algorithm-specific misclassification estimates are used as informative priors, enabling calibration without direct access to CHAMPS data (see online supplemental sections S1.6 and 3.8 in Pramanik *et al*^30^). The effectiveness of VA-calibration depends on the structure of misclassification. While low individual-level sensitivity poses challenges for VA, calibration can compensate for this, provided the misclassification matrices remain non-singular, that is, the CCVA algorithm exhibits systematic and sufficiently distinct misclassification patterns across causes. Under this condition, the calibration equation *q*=*Φ*^*T*^*p*, which relies on inversion of the misclassification matrix (*Φ*) to recover the true cause distribution (*p*), remains well posed. Figure 2 outlines the calibration pipeline. We do not calibrate for the ‘other’ cause as it comprises different causes in VA and CHAMPS diagnoses. Accordingly, the misclassification matrices are renormalized to ensure they remain valid.

#### Ensemble calibration

CCVA algorithms often disagree in predicting COD, making it challenging to identify the most accurate algorithm. Following *Datta et al.*^26^ and Fikse *et al.,*^27^ we perform ensemble VA-calibration by integrating misclassification rates of all algorithms in a Bayesian model. This lowers the risk of using an inaccurate algorithm and improves CSMF estimates.

## Results

We present VA misclassification analysis using CHAMPS data and apply Mozambique-specific estimates to COMSA-Mozambique. Results for children are summarised briefly, with full details in online supplemental section S3.

### VA misclassification estimates from CHAMPS analysis

#### Neonatal deaths (aged 0–27 days)

##### VA misclassification rates and model comparison

**Figure 3:**
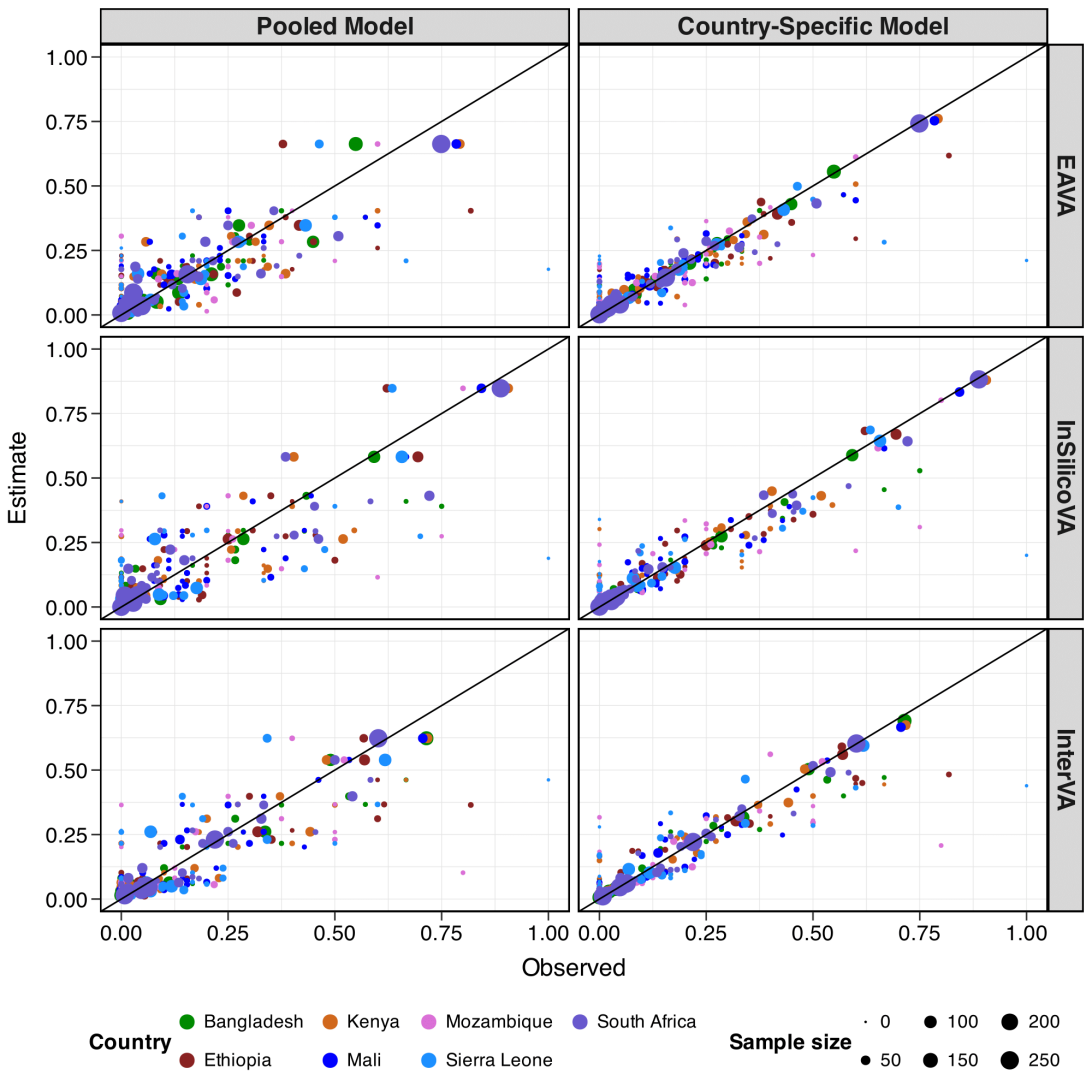
Scatterplots of observed (x-axis) and estimated (y-axis) misclassification rates of Expert Algorithm VA (EAVA^4^) (top row), InSilicoVA^5^ (middle row) and InterVA^6^ (bottom row) for neonatal deaths (aged 0–27 days) in CHAMPS. It compares estimates from the homogeneous or pooled model (left panels) and the country-specific model (right panels). Countries are denoted in different colours. The point size reflects the observed sample size for corresponding CHAMPS cause. The black line corresponds to the line of equality *y*=*x*. Compared with the homogeneous model, the country-specific model reduces the average absolute loss with respect to observed rates by 35%, 38% and 34% for the three algorithms for this age group. CHAMPS, Child Health and Mortality Prevention Surveillance; VA, verbal autopsy.

Based on 1379 neonatal records from CHAMPS, figure 3 compares observed misclassification rates with estimates from the homogeneous (left) and country-specific (right) models for the three algorithms. Two key insights emerge: first, the country-specific estimates align more closely with the observed rates, as indicated by their proximity to the line of equality (*y*=*x*), where *x* represents the observed rates and *y* represents the corresponding model-based estimates. Second, in the country-specific model, larger points (more samples) are closer to the *y*=*x* line, reflecting the model’s greater reliance on observed rates under sufficient samples. The few points that deviate from the *y*=*x* line in the country-specific model reflect a low sample size. In that case, the model shrinks towards the pooled estimate, causing deviations from observed rates. To quantify the improvement, we calculate the average absolute difference between the modelled estimate ${\tilde{\Phi }}_{s}=\left({\tilde{\phi }}_{sij}\right)$ and the observed rate ${\hat{\Phi }}_{s}=\left({\hat{\phi }}_{sij}\right)$ across all countries and cause pairs, using the absolute loss function $\sum_{sij}|{\tilde{\phi }}_{sij}-{\hat{\phi }}_{sij}|/Obs$, where Obs is the total number of observed CHAMPS-VA cause pairs. Online supplemental figures S3, S4 and S7–S9 compare observed and estimated rates for both models. Online supplemental figure S12 presents effect size estimates highlighting evidence of pull and cross-country heterogeneity.

Online supplemental figure S4 presents the estimated country-specific misclassification rates. The leftmost column shows rates pooled across countries, highlighting substantial variation in sensitivity by cause and across CCVA algorithms. Sensitivity is highest for prematurity (ranging from 62% to 85%) and lowest for sepsis/meningitis/infection (12%–22%) and ‘other’ (5%–10%). The performance also varies across algorithms: InSilicoVA has difficulty detecting congenital malformations, while EAVA and InterVA achieve sensitivities of about 41% and 35%, respectively. Pooled false negative rates are notable as well; for example, 31%–44% of sepsis/meningitis/infection deaths are misclassified as prematurity, and 12%–39% of congenital malformation deaths are misclassified in the same way, depending on the algorithm.

Relative to the homogeneous model, the country-specific model improved point estimates for 72%–91% of cause pairs, reducing 44%–50% absolute bias on average. For EAVA, InSilicoVA and InterVA, the country-specific model reduced average absolute loss by 35%, 38% and 34%, respectively. Additionally, it enhanced uncertainty quantification for 62%–70% of cause pairs, with 75%–79% reductions on average in interval scores (see online supplemental figures S10 and S11).

##### Estimate of intrinsic accuracy and pull

Online supplemental figure S13 illustrates estimates of intrinsic accuracy and pull, which serves as diagnostics of CCVA algorithms. All algorithms have the highest intrinsic accuracy for prematurity, with InSilicoVA performing best for this cause. The accuracy is the lowest for ‘other’. Regarding pull, no preference for an algorithm indicates a uniform pull of 1/6 for six causes, with deviations from 1/6 suggesting a systematic bias. EAVA favours pneumonia but underpredicts congenital and ‘other’; InSilicoVA overpredicts prematurity and underpredicts congenital and ‘other’; and InterVA favours IPRE and prematurity while down-weighting other causes. These diagnostics reveal algorithm-specific tendencies.

##### Examining the sources of heterogeneity in VA misclassification

The left panel in online supplemental figure S5 highlights variability in EAVA’s sensitivity for sepsis/meningitis/infection, which varies from 6% (South Africa) to 31% (Kenya). This is partly due to EAVA’s hierarchical structure, where sepsis ranks low, only above jaundice, neonatal haemorrhage and sudden unexplained infant death, which are categorised as ‘other’. Among 61 deaths with CHAMPS cause sepsis/meningitis/infection, 37 met clinical criteria for sepsis by EAVA, and only two were assigned sepsis. The other 35 records have an EAVA cause, in addition to sepsis, which is higher up in the hierarchy: 2 congenital malformations, 8 IPREs, 12 pneumonia and 13 prematurity.

The right panel of the figure shows higher false-negative rates for the CHAMPS-VA cause pair IPRE-prematurity in Kenya and South Africa, with all records meeting EAVA’s prematurity criteria. IPRE (due to birth injury or asphyxia) ranks in the top third of the hierarchy, above prematurity. Diagnosing birth asphyxia requires answering the VA question id10106 (‘How many minutes after birth did the baby first cry?’), which is missing in 7 of 20 deaths in Kenya and 11 of 17 deaths in South Africa. This highlights the need to improve data quality through better tools and interviewer training.

### Summary of VA misclassification rates for children (aged 1–59 months)

Analysis of VA misclassification for 1080 child deaths in CHAMPS shows significant variability across causes, algorithms, and countries (online supplemental figures S16–S20). Overall, InSilicoVA and InterVA have very low sensitivity for neonatal causes (3%–4%), while EAVA’s sensitivity is relatively higher (37%). False negative rates are substantial, especially for malaria and other infections (25%–30%), with notable country-level variation.

The country-specific misclassification model outperforms the homogeneous model by reducing absolute bias in 69%–72% of cause pairs with 27%–40% bias reduction on average, and improving uncertainty quantification with 56%–68% lower interval scores in 65%–69% of cause pairs. For children, it also lowers average absolute loss by 19%, 24% and 13% for EAVA, InSilicoVA and InterVA. Detailed results are in online supplemental section S3.1 and figures S14–S29.

### Case study: CSMF estimates in Mozambique using COMSA-Mozambique data

#### Neonates (aged 0–27 days)

##### Misclassification estimates from CHAMPS as prior information

**Figure 4:**
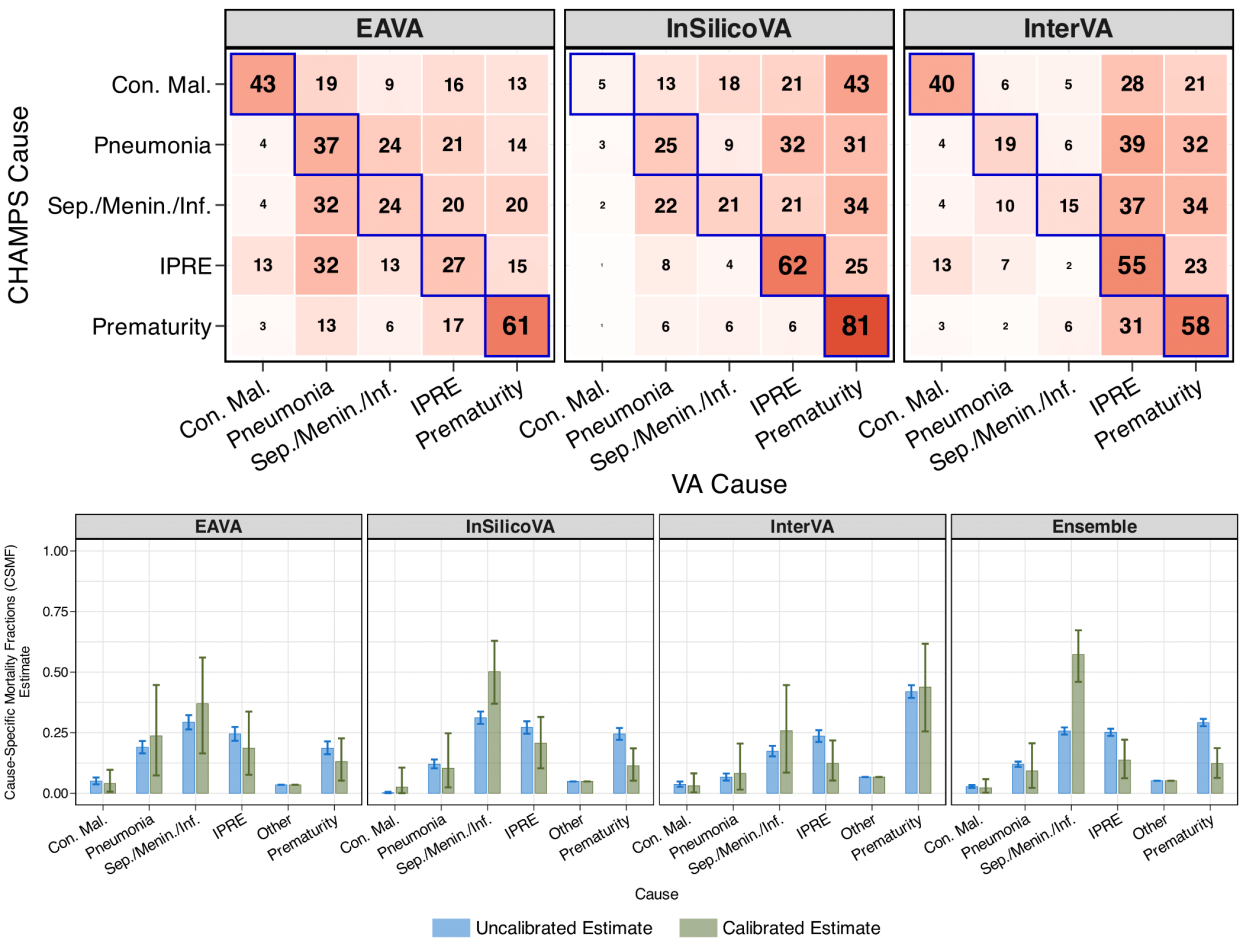
Top panel: obtained from CHAMPS analysis of neonatal (aged 0–27 days) deaths, this is the (expected) misclassification (without ‘other’) for Mozambique that is used as an informative prior in modular VA-calibration. Sensitivities are along diagonals (outlined in blue). Bottom panel: comparison of uncalibrated (blue) and calibrated (green) CSMF estimates for neonates in Mozambique using EAVA^4^, InSilicoVA^5^, InterVA^6^ and their ensemble. Bar heights represent the point estimates (posterior means), while the error bars indicate uncertainty (95% credible intervals). Although uncalibrated CSMF estimates have lower uncertainty than calibrated ones, they assume perfect classification, an assumption contradicted by substantial VA misclassification observed in CHAMPS. No calibration can lead to overconfident and biased CSMF estimates. CHAMPS, Child Health and Mortality Prevention Surveillance; Con mal, congenital malformation; CSMF, cause-specific mortality fraction; EAVA, Expert Algorithm VA; IPRE, intrapartum-related events; Sep/Menin/Inf, sepsis/meningitis/infection; VA, verbal autopsy.

The top panel of figure 4 presents the (expected) Mozambique-specific misclassification estimates from analysing CHAMPS data. They are used as an informative prior for modular VA-calibration of VA-only COD data from COMSA-Mozambique. Sensitivities are generally high for prematurity (particularly in InSilicoVA) and IPRE (except EAVA). False negatives are evident across all algorithms, especially for VA causes IPRE and prematurity.

##### Raw CSMF estimates

The bottom panel of figure 4 presents raw neonatal CSMF estimates (blue) in Mozambique from VA-only data. Among 1192 deaths, EAVA and InSilicoVA identify sepsis/meningitis/infection as the leading cause, while InterVA and the ensemble rank prematurity highest. All algorithms consistently list IPRE as the second most common cause and congenital malformations as the least common.

##### Calibrated CSMF estimates

The bottom panel of figure 4 shows uncertainty-quantified calibrated CSMF estimates (green). Calibration increases the CSMF for sepsis/meningitis/infection and decreases it for IPRE and prematurity (except for InterVA). These reflect misclassifications, where algorithms misclassify 20%, 21% and 37% of sepsis/meningitis/infection deaths as IPRE, and 20%, 34% and 34% of sepsis/meningitis/infection deaths as prematurity (see figure 4). The calibration adjusts for the undercounting of sepsis/meningitis/infection deaths and overcounting of IPRE and prematurity.

The far-right comparison in the same panel shows ensemble-calibrated CSMF estimates, with calibration attributing 58% of neonatal deaths to sepsis/meningitis/infection, followed by 13% to IPRE, 12% to prematurity, and smaller shares to other causes. The 95% credible intervals confirm an increase in sepsis/meningitis/infection (47%–68% vs 26% uncalibrated) and a decrease in prematurity and IPRE (6%–18% and 6%–22% vs 29% and 25% uncalibrated). Calibrated intervals for other causes overlap with uncalibrated values. These align with previous findings in Fiksel *et al*.^28^

### Children (aged 1–59 months)

#### Misclassification estimates from CHAMPS

**Figure 5:**
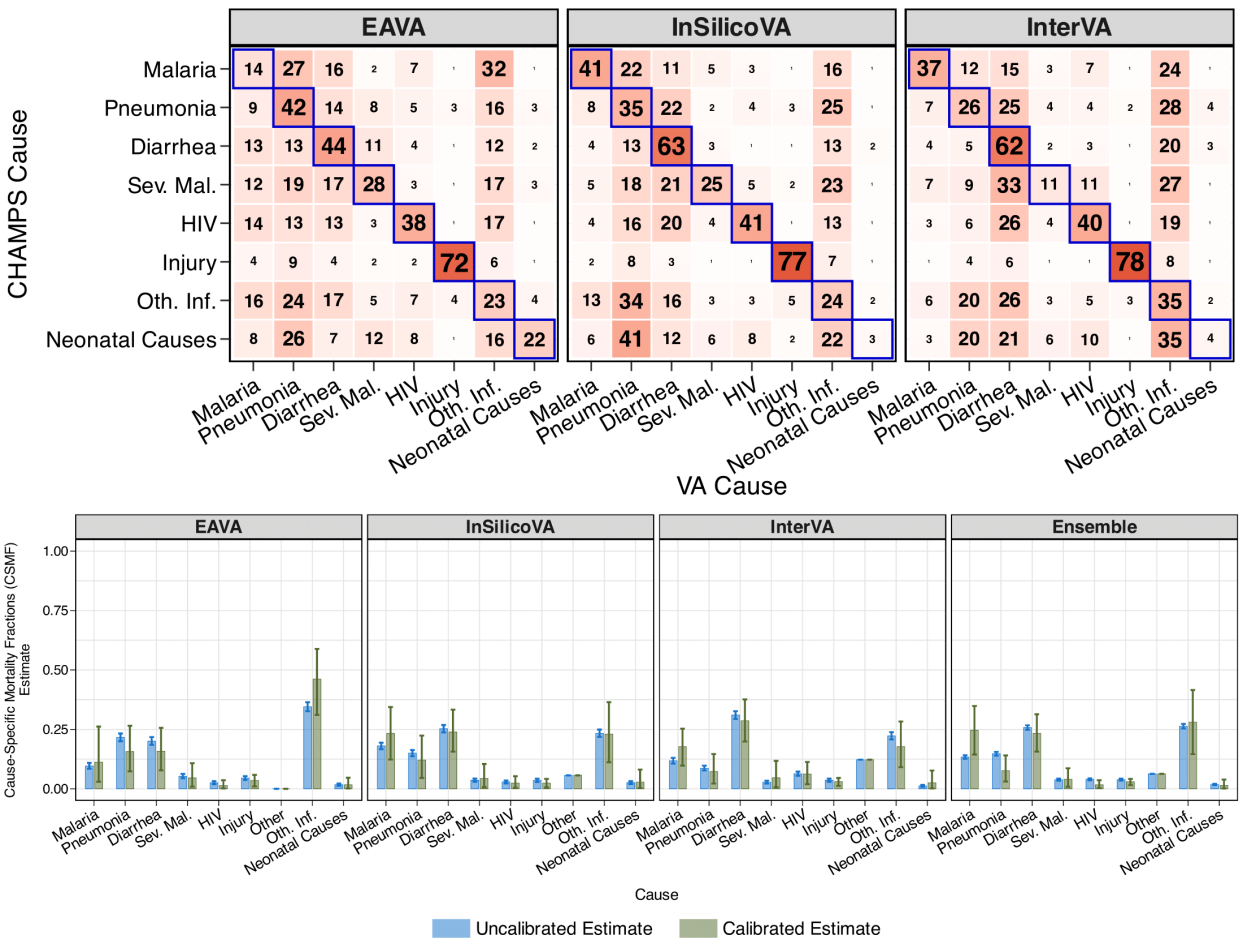
Top panel: obtained from CHAMPS analysis of child (aged 1–59 months) deaths, this is the (expected) misclassification (without ‘other’) for Mozambique that is used as an informative prior in the modular VA-calibration. Sensitivities are along diagonals (outlined in blue). Bottom panel: comparison of uncalibrated (blue) and calibrated (green) CSMF estimates for children using EAVA^4^, InSilicoVA^5^, InterVA^6^ and their ensemble. Bar heights represent the point estimates (posterior means), while the error bars indicate uncertainty (95% credible intervals). Although uncalibrated CSMF estimates show lower uncertainty than calibrated ones, they assume perfect classification, an assumption contradicted by substantial VA misclassification observed in CHAMPS. No calibration can lead to overconfident and biased CSMF estimates. CHAMPS, Child Health and Mortality Prevention Surveillance; CSMF, cause-specific mortality fraction; EAVA, Expert Algorithm VA; Oth Inf, other infections; Sev mal, severe malnutrition; VA, verbal autopsy.

For the three CCVA algorithms, the top panel of figure 5 shows (expected) Mozambique-specific misclassification estimates from analysing CHAMPS data. Sensitivity is the highest for injury and diarrhoea, with high false negatives for VA causes pneumonia, diarrhoea and ‘other infections.’

#### CSMF estimates

The bottom panel of figure 5 compares uncalibrated (blue) with calibrated (green) CSMF estimates for 2812 VA-only child deaths from COMSA-Mozambique. Calibration generally increases the estimated CSMF for malaria and decreases it for pneumonia and diarrhoea. The 95% credible intervals typically include the uncalibrated estimates but lie near the edges for some causes, especially other infections (EAVA), malaria (InterVA and ensemble), and pneumonia (ensemble). Overall, the findings, particularly for malaria and pneumonia, align with results from prior research.^28^ Detailed results are in online supplemental section S3.2. In online supplemental tables S1 and S2 in Section S4, we present Mozambique’s age-group-specific and algorithm-specific CSMF estimates featured in figures 4 and 5.

## Discussion

Using CHAMPS data, we estimated misclassification matrices for EAVA, InSilicoVA and InterVA, broken down by eight countries (Bangladesh, Ethiopia, Kenya, Mali, Mozambique, Sierra Leone, South Africa, and ‘other’) and two age groups (neonate: aged 0–27 days; children: aged 1–59 months). These estimates, derived using the country-specific misclassification model,^30^ significantly outperformed a homogeneous model by reducing bias and improving uncertainty quantification. A reanalysis of COMSA-Mozambique’s VA-only data using Mozambique-specific misclassification estimates demonstrates the practical benefit. Methodologically, our findings highlight the critical need to account for heterogeneity and systematic preference in CCVA misclassification, which, if unaccounted for, can obscure important differences driven by epidemiology, health systems and algorithm design. Our research offers a principled way to improve CSMF estimation accuracy by developing country-specific misclassification matrices and integrating them into a rigorous calibration framework.

This study’s findings are vital for estimating population-level mortality in LMICs. While MITS and the CHAMPS DeCoDe process provide high-quality COD data, their scalability at the population level is constrained by cost and feasibility. In contrast, VA remains the only viable population-representative method in low-resource settings. We show VA can still support accurate population-level inferences, despite low individual-level accuracy (algorithmic sensitivities often <50%; see online supplemental figures S4 and S17), provided misclassification patterns are systematic and characterisable, and the resulting misclassification matrix is sufficiently non-singular (see online supplemental figure S30).^26 27 30^ To facilitate this, we have publicly released the misclassification matrices and the ‘vacalibration’ R package (GitHub, CRAN), and integrated it into openVA, a leading VA-based COD analysis software. This eliminates the need for gold-standard COD data like MITS for VA-only analysis. As CHAMPS expands and VA surveillance advances (eg, Zhu and Li^31 32^ and Kunihama *et al*^31^,^33^), our work provides a practical approach for immediately strengthening existing VA systems and a framework readily adaptable for ongoing innovations.

VA-calibration’s efficacy is predicated on the transportability assumption that the misclassification in labelled data (eg, CHAMPS) applies to unlabelled VA data (eg, COMSA-Mozambique). This is unverifiable in the absence of reference causes for unlabelled data and assumes globally consistent VA symptoms given a true cause. High misclassification rates also show the limitation of single-cause attribution, as many under-five deaths involve interacting conditions, meaning VA may capture intermediate causes in the causal chain. This motivates a multi-cause framework that assigns probabilistic weights to causes.^27 29 34^ VA calibration also assumes temporal stability in misclassification, an assumption challenged by real-world variability in disease prevalence, healthcare access and VA implementation. Future research will address cross-country differences and algorithmic bias in these settings, aiming to align VA surveillance with the multifactorial nature of child mortality.

Accurate COD data are crucial for strengthening health systems and informing national policies and investments. Our work highlights both the need and the opportunity to improve existing VA-calibration, which will, in turn, reinforce the role of VA-based mortality surveillance. Together, VA and MITS form a complementary system: MITS offers diagnostic precision and depth, while VA provides breadth and scalability. Sustained investment in CHAMPS, integration of MITS-informed calibration into VA workflows and ongoing methodological innovation are vital. These steps will enable within-country calibration, improving accuracy, especially where mortality patterns vary significantly geographically. Additionally, countries with comparable epidemiological profiles (eg, similar mortality levels, HIV prevalence and malaria burden) can benefit from leveraging each other’s MITS data for calibration when local data is scarce. More recently, CCVA algorithms based on new techniques (eg, latent class models, domain adaptation, machine learning and large language models) with higher individual-level accuracy^910 31^,^33 35^ and AI-driven initiatives such as the COD Assistance are promising advances for both primary VA data collection and automated COD assignment. However, structured misclassification will likely persist. This underscores the timeliness and relevance of our presented VA-calibration workflow, which is well-suited to adapt to continuous methodological improvements and advance a more accurate, equitable and actionable system for global mortality surveillance.

## Supplementary material

10.1136/bmjgh-2025-021747 online supplemental file 1

10.1136/bmjgh-2025-021747 online supplemental file 2

## Data Availability

Data are available upon reasonable request.
