# Supplementary material for: Country-specific estimates of misclassification rates of computer-coded verbal autopsy algorithms
Source: BMJ Glob Health. 2026 Mar 9;11(3):e021747. doi: 10.1136/bmjgh-2025-021747 (PMC12983874; doi:10.1136/bmjgh-2025-021747)
Supplement: online supplemental file 1 [file bmjgh-11-3-s001.docx]

### BMJ Global Health Author Reflexivity Statement

Adapted from Morton, B., Vercueil, A., Masekela, R., Heinz, E., Reimer, L., Saleh, S., Kalinga, C., Seekles, M., Biccard, B., Chakaya, J., Abimbola, S., Obasi, A. and Oriyo, N. (2022), Consensus statement on measures to promote equitable authorship in the publication of research from international partnerships. Anaesthesia, 77: 264-276. <https://doi.org/10.1111/anae.15597>

| **Study conceptualisation** | |
| --- | --- |
| 1. How does this study address local research and policy priorities? | This is a secondary data analysis. Please see our response to item 5 below.  The findings will help communities participating in the Child Health and Mortality Prevention Surveillance (CHAMPS) understand the reliability of verbal autopsy (VA) and how the post-mortem diagnostics used in CHAMPS add useful information to improve health programs.  The study findings respond Mozambique’s health program and policy decisionmakers demand for improved and accurate information of cause of death (COD) distribution in children under age five. The study capacitates local research partners in conducting calibrated VA analyses. |
| 1. How were local researchers involved in study design? | This is a secondary data analysis. Please see our response to item 5 below.  The leaders of each site in the CHAMPS network and their research teams all contribute to the design of CHAMPS data collection protocols and performed participant enrolment, specimen testing, and data collection. They also approved use of the CHAMPS data for the analysis presented in this manuscript.  In Mozambique, the National Institute of Health (INS) leads the implementation of the COMSA surveillance initiative and the local PI is involved in this secondary study. |
| **Research management** | |
| 1. How has funding been used to support the local research team(s)? | This is a secondary data analysis. Please see our response to item 5 below.  The work of the CHAMPS Network is supported by funding from the Gates Foundation. This funding covers salaries for local research teams in addition to the local infrastructure that is needed for the work.  In Mozambique, JHU provides continued technical assistance to the local research team at the National Institute of Health on the implementation of the COMSA surveillance and builds the capacity of the local researchers. |
| **Data acquisition and analysis** | |
| 1. How are research staff who conducted data collection acknowledged? | This is a secondary data analysis. Please see our response to item 5 below.  In CHAMPS, the leaders of each site and their research teams all contribute to the design of CHAMPS data collection protocols and performed participant enrolment, specimen testing, and data collection. They also approved use of the CHAMPS data for the analysis presented in this manuscript.  In Mozambique, the National Institute of Health (INS) leads the implementation of the COMSA surveillance initiative and the local PI is involved in this secondary study.  CHAMPS representatives and local PI of COMSA-Mozambique are authors of this manuscript. |
| 1. How have members of the research partnership been provided with access to study data? | We are using unidentifiable COD data that are collected by the CHAMPS Network. We use the anonymized VA-COD data collected in the Comprehensive Mortality Surveillance for Action (COMSA) program in Mozambique (COMSA-Mozambique; OPP1163221; PI: Drs. Amouzou and Macicame). This is considered nonhuman Secondary subjects research under HHS human subject regulation (45 CFR Part 46). The data used in this research, are already collected in COMSA-Mozambique and CHAMPS, and the authors only perform statistical analyses with them.  Data collected and generated at CHAMPS sites are de-identified and submitted to a central database, housed at Emory University. Each CHAMPS site has signed a data use agreement (DUAs) with Emory that allows this data transfer and aggregation. In addition, the DUAs signed by CHAMPS sites allow Emory University to share the aggregated dataset with other researchers, once they sign an appropriate DUA with Emory. This process was how the data were provided from CHAMPS for this analysis.  In Mozambique JHU has access to the COMSA data through its collaboration with INS for the implementation of the surveillance initiative. |
| 1. How were data used to develop analytical skills within the partnership? | This is a secondary data analysis. Please see the response to item 5 above.  The authors used the data in analytic planning, running and reviewing code outputs, interpreting results, and iterative quality checks to strengthen analytical skills across the team.  Also, in Mozambique, the COMSA system include a collaborative analysis web portal through which study investigators collaborate on data analysis, sharing of statistical codes and results. |
| **Data interpretation** | |
| 1. How have research partners collaborated in interpreting study data? | This is a secondary data analysis. Please see the response to item 5 above.  Authors and representatives from CHAMPS and COMSA-Mozambique meet monthly to participate in the data analysis and interprete the findings. Final products are already reviewed by them. |
| **Drafting and revising for intellectual content** | |
| 1. How were research partners supported to develop writing skills? | This is a secondary data analysis. Please see the response to item 5 above.  No additional writing-skills training or support was provided beyond routine collaborative manuscript development (shared outlining, drafting, and iterative co-editing by all partners).  In Mozambique, COMSA project organized data analysis and writing workshops with local partners. They have been involved and led many write up in the COMSA supplement published in the [AJTMH](https://www.ajtmh.org/view/journals/tpmd/108/5_Suppl/tpmd.108.issue-5_Suppl.xml). |
| 1. How will research products be shared to address local needs? | To facilitate broader utility of findings from this research, we have made the inventory of misclassification matrix estimates (learned from the CHAMPS data) publicly available on [GitHub](https://github.com/sandy-pramanik/CCVA-Misclassification-Matrices) and have released the ‘vacalibration’ R package on both [CRAN](https://cran.r-project.org/web/packages/vacalibration/index.html) and [GitHub](https://github.com/sandy-pramanik/vacalibration). The VA-calibration framework is also [fully integrated](https://cran.r-project.org/web/packages/openVA/vignettes/vacalibration-vignette.html) into [openVA](https://cran.r-project.org/web/packages/openVA/index.html), a leading platform for VA-based COD analysis.  In Mozambique, the INS disseminates regularly findings from the COMSA project to the Ministry of Health and stakeholders. |
| **Authorship** | |
| 1. How is the leadership, contribution and ownership of this work by LMIC researchers recognised within the authorship? | This is a secondary data analysis. Please see our response to item 5 above.  LMIC researchers’ leadership and contributors are recognised here as authors that reflects substantive intellectual input (study conception, interpretation, drafting/revision). |
| 1. How have early career researchers across the partnership been included within the authorship team? | This is a secondary data analysis. Please see our response to item 5 above.  Early career researchers are recognised here as authors reflecting their substantive contributions to analysis, interpretation, and/or drafting and revision of the manuscript. |
| 1. How has gender balance been addressed within the authorship? | This is a secondary data analysis. Please see our response to item 5 above.  We sought to ensure gender balance within the authorship by inviting eligible contributors of all genders across partner institutions and finalising the author list based on substantive contributions. |
| **Training** | |
| 1. How has the project contributed to training of LMIC researchers? | This is a secondary data analysis. Please see our response to item 5 above.  CHAMPS has provided training opportunities for students and young researchers, and continuing education opportunities for healthcare workers.  In Mozambique, the COMSA project has trained multiple researchers at INS on data collection, review, and analysis. They also serve as co-author on publications using these data. |
| **Infrastructure** | |
| 1. How has the project contributed to improvements in local infrastructure? | This is a secondary data analysis. Please see our response to item 5 above.  The findings reported in this manuscript have been made publicly available (see our response to item 9 above).  Investments in CHAMPS have built local capacity to perform research and perform laboratory assays. In addition, findings from CHAMPS have led to local improvements in some aspects of the healthcare system.  In Mozambique, the COMSA project support local infrastructure at central (Maputo) and provincial level, including IT, software, offices, vehicles, and logistics needed for data collection, analysis, and dissemination. |
| **Governance** | |
| 1. What safeguarding procedures were used to protect local study participants and researchers? | This is a secondary data analysis. Please see our response to item 5 above.  The findings presented in this manuscript are reported as population-level aggregates and are not identifiable at the individual participant-level.  Enrollment in CHAMPS only takes place after conducting informed consent with parents of the deceased. Identifying information is kept confidential. The study protocol is reviewed by ethics committees for each CHAMPS site and Emory University. In addition, CHAMPS sites have grief counselling for researchers, given the challenging nature of working with families who have lost children, and all sites follow local labor protection laws.  COMSA is a sample mortality surveillance system in which participants consent to participate. Data collection is not invasive and VA data collectors are trained to be sensitive to family grief and refer the family for counseling in cases of distress. |
